# Transformation to estimate the causal effect in Mendelian randomization study with binary risk factor and outcome

**DOI:** 10.1186/s12859-026-06388-1

**Published:** 2026-02-27

**Authors:** Nesma Lotfy

**Affiliations:** Department of Biostatistics, High Institute of Public Health, Alexandria University, Alexandria, Egypt

**Keywords:** Mendelian randomization, Binary, Wald ratio, Two-stage predictor substitution, Two-stage residual inclusion, Simulation

## Abstract

**Background:**

In some GWAS studies, particularly those involving biobank data, linear regression is employed to obtain summary statistics on binary traits, while others report the log odds or odds ratios from the logistic regression of the genomic variants. However, some studies applied a transformation equation between logistic regression to linear regression.

**Aim:**

The current study aims to assess the performance of the Wald ratio using logistic regression, linear probability models (LPM), and transformation approaches in comparison with structural equation modelling (SEM) and Two Stage Predictor Substitution (TSPS), Two Stage Residual (TSRI) via simulation and real data analysis.

**Methods:**

Simulation data based on a bivariate Bernoulli distribution were applied within an instrumental variable framework to estimate empirical bias. Four sensitivity analysis scenarios were considered, varying the sample size, IV prevalence, exposure and outcome prevalence, and confounder effect. Additionally, real data from the Golden Retriever Lifetime Study were analyzed to estimate the potential causal effect of activity level on cancer risk.

**Results:**

In the simulation data, for the positive effect size with a low confounder effect and a weak instrumental variable scenario, the median (Q1–Q3) biases of the Wald ratio were as follows: under SEM, the bias for logistic regression was 0.77 (−0.20–1.74), for LPM it was 0.03 (−3.78–3.95), and for the transformation method it was 0.03 (−3.79–3.94). Under TSPS, the bias for logistic regression was 0.72 (−2.24–3.61), for LPM it was 0.00 (−0.04–0.05), and for the transformation method it was 0.00 (−0.01–0.01). Under TSRI, the bias for logistic regression was 0.75 (−2.47–3.87), for LPM it was 0.02 (−0.21–0.26), and for the transformation method it was 0.02 (−0.22–0.26). In the real data analysis, for the SNP Affx-205724246_A, the biases of the Wald ratio were as follows: under SEM, −0.14 for logistic regression, −1.30 for LPM, and −1.45 for the transformation method. Under TSPS and TSRI, the bias for logistic regression was 1.24, for LPM 0.08, and for the transformation method −0.07.

**Conclusion:**

The findings indicated that increasing the strength of the instrumental variable led to a reduction in bias, with the best performance observed at instrumental strengths of 0.5 and 0.7. The LPM and transformation approaches produced relatively lower bias in the TSPS framework when the confounder effect was below 0.1 and the prevalence of the outcome or the exposure is within 0.5 to 0.62. When the prevalence of the exposure and outcome ranged from 0.67 to 0.84 and the instrumental strength was high (0.7), the bias of the LPM and transformation methods were slightly higher but comparable to that observed when the prevalence ranged from 0.5 to 0.62. Furthermore, low prevalence of exposure or outcome (ranged from 0.12 to 0.23) produced larger bias than those observed at higher prevalence values (ranged from 0.67 to 0.84). In the real data analysis, the bias of the Wald ratio using LPM and transformation methods were lower under TSPS and TSRI and higher under the SEM method, while the bias of the Wald ratio using logistic regression was lower under SEM compared to the other methods.

**Supplementary Information:**

The online version contains supplementary material available at 10.1186/s12859-026-06388-1.

## Introduction

A Genome-Wide Association Study (GWAS) is a research approach used to identify genomic variants that are statistically associated with a risk for a disease. A GWAS database serves as a repository for the results of GWAS studies, allowing researchers to access the data for further analysis. Mendelian randomization (MR) uses genetic variants as instrumental variables to estimate the causal effects of exposure (risk factor) on an outcome using observational data [1, 2]. As GWAS summary statistics become more widely available, two-sample Mendelian Randomization (MR), which utilizes independent GWAS datasets for exposure and outcome, has gained widespread popularity for conducting MR studies [3]. In two-sample Mendelian Randomization, a key assumption is that genetic variants have a linear relationship with both exposure and outcome, allowing the causal effect to be estimated as the ratio of the variant-outcome association to the variant-exposure association, provided there is no horizontal pleiotropy (Wald ratio) [4]. However, when either the exposure or outcome traits are binary, the effects are estimated on the odds ratio scale, it is no longer possible to reliably calculate the causal effects because of the noncollapsibility of odds ratios even under no horizontal pleiotropy [5, 6]. A simulation study was conducted to assess the bias of the Wald ratio when the exposure and outcome binary. It was found that under a null causal effect, the Wald method demonstrated good accuracy. However, with a positive causal effect, the method exhibited persistent bias toward the true value [7]. Several methods are available to estimate the causal effect of an exposure on an outcome using instrumental variables, including Two Stage Predictor Substitution (TSPS) [7], Two Stage Residual (TSRI) [7], and Structural Equation Modelling (SEM) [8].

Marginal effects measure the impact that an instantaneous change in one variable has on the outcome variable while all other variables are held constant. It plays a crucial role in interpreting the relationship between explanatory variables and the dependent variable. In ordinary least squares regression with no interactions or higher-order terms, the estimated slope coefficients are marginal effects. However, in logistic regression, the probability of an outcome is modelled using a nonlinear transformation (logistic function). This transformation makes the direct interpretation of coefficients more challenging, which is why marginal effects are widely used to quantify the impact of independent variables on the probability of an outcome. The Linear Probability Model (LPM) is a simple and widely used approach for estimating marginal effects in binary outcome models. LPM applies ordinary least squares (OLS) to estimate the probability of an event occurring as a linear function of the explanatory variables. A key issue with LPM is that the errors are heteroscedastic. To address this, a two-step weighted least squares approach proposed by Goldberger (1964) [9] yields estimators that are unbiased, efficient and asymptotically normally distributed, allowing hypotheses about the true coefficient values to be tested using standard methods. Another major issue is that the predicted probabilities can lie outside the [0, 1] interval. This drawback is the main reason the LPM has fallen out of favor, with Logit and Probit models becoming more commonly used for binary regression [10].

In some GWAS studies, particularly those involving biobank data, linear regression is employed to obtain summary statistics on binary trait, while others report the log odds or odds ratios from the logistic regression of the genomic variants. Some studies applied a Transformation equation between logistic regression to linear regression. Therefore, the current study aims to assess the performance of the Wald ratio using logistic regression, linear probability models (LPM), and transformation approaches in comparison with structural equation modelling (SEM) and two-stage estimation methods.

## Methods

The transformation method to convert logistic regression coefficient to linear probability regression coefficient [11].

### Transformation equation

$$ \beta_{linear} = \beta_{logistic} *\left( {\left( {pr} \right)*\left( {1 - pr} \right)} \right) $$

where pr is the probability of binary trait (outcome).

### Estimation Mendelian randomization causal effect

In two sample Mendelian randomization studies, the causal effect can be estimated through Wald ratio using:

#### Logistic regression

This method proceeds by fitting two logistic regressions.

$$ {\mathrm{Logit}}\,(P_{X} ) = \alpha_{0} + \alpha_{1} * Z $$

$$ {\mathrm{Logit}}\,(P_{Y} ) = \gamma_{0} + \gamma_{1} * Z $$

The Wald method causal effect estimator is then formed as the ratio of the estimated.

slope parameters

$${\beta }_{Wald}=\frac{{\gamma }_{1}}{{\alpha }_{1}}$$

#### Linear probability model (LPM)

Conduct linear probability model for both models

$$ {\mathrm{X}} = \alpha_{0} + \alpha_{1} * Z $$

$$ {\mathrm{Y}} = \gamma_{0} + \gamma_{1} * Z $$

$${\beta }_{LPM.trans}= \mathrm{transformation}^{-1} (\frac{{\gamma }_{1}}{{\alpha }_{1}}),$$

where transformation^−1^ defined as:

$${\beta }_{logistic}={\beta }_{linear}/(\left(pr\right)*\left(1-pr\right))$$

#### Transformation method

This method proceeds by fitting two logistic regressions.

$$ {\mathrm{Logit}}\,(P_{X} ) = \alpha_{0} + \alpha_{1} * Z $$

$$ {\mathrm{Logit}}\,(P_{Y} ) = \gamma_{0} + \gamma_{1} * Z $$

Next, the coefficients ${\alpha }_{1}$ and ${\gamma }_{1}$ are transformed from the log-odds scale to the linear.

scale using the transformation equation, after which the Wald ratio is calculated as:

$${\beta }_{linear}=\frac{{\gamma }_{transformed.linear}}{{\alpha }_{transformed.linear}}$$

The resulting ${\beta }_{linear}$ is then converted back to the log-odds scale using the inverse.

transformation (transformation^-1^):

$${\beta }_{tranformed}=\text{ transformation}^{-1} ({\beta }_{linear})$$

where transformation^−1^ defined as:

$${\beta }_{logistic}={\beta }_{linear}/(\left(pr\right)*\left(1-pr\right))$$

### Instrumental variable methods

The causal effect can be estimated through.

#### Structural estimation equation (SEM)

The SEM approach allows researchers to specify a system of equations that represents the causal structure, it can be implemented using maximum likelihood. The equations will be

$$ {\mathrm{Logit}}\,(P_{X} ) = \alpha_{0} + \alpha_{1} * Z $$

$$ {\mathrm{Logit}}\,(P_{Y} ) = \beta_{0} + \beta_{sem} * X $$

where ${\beta }_{sem}$ represent the causal effect between X and Y.

#### Two stage predictor substitution (TSPS) [7]

The first‐stage regression

$$ {\mathrm{Logit}}\,(P_{X} ) = \alpha_{0} + \alpha_{1} * Z $$

Subsequently, $\widehat{{P}_{x}}$ is plugged into the second‐stage equation to estimate the causal effect of interest ${\beta }_{TSPS}$. The second stage regression

$$ {\mathrm{Logit}}\,(P_{Y} ) = \beta_{0} + \beta_{TSPS} * \hat{P}_{x} $$

#### Two stage residual (TSRI) [7]

Similarly to TSPS, TSRI involves a two‐stage system of logistic regressions in this setting.

The first‐stage regression

$$ {\mathrm{Logit}}\,(P_{X} ) = \alpha_{0} + \alpha_{1} * Z $$

$$ \hat{ \in }_{x} = {\text{X - }}P_{X} $$

Subsequently, $\widehat{{\upepsilon }_{x}}$ is plugged into the second‐stage equation to estimate the causal effect of interest ${\beta }_{TSRI}$. The second stage regression

$$ {\mathrm{Logit}}\,(P_{Y} ) = \beta_{0} + \beta_{TSRI} * X + \gamma \hat{ \in }_{x} $$

### Simulation

To evaluate the practical performance of the Wald ratio methods, we generated multiple simulated datasets to compare the methods described above when using a single instrumental variable (IV). The simulations involved binary variables for the instrument (Zi), exposure (Xi), and outcome (Yi), with the association between X and Y confounded by a normally distributed variable Ui with a mean of zero. The IV (Zi) was drawn from a Bernoulli distribution with a probability (pZ) of 0.2 across all scenarios. The confounder (Ui) followed a standard normal distribution N(0, 1). The exposure variable (Xi) was generated from a Bernoulli distribution with a probability defined as px = expit(α₀ + α₁ * Zi + cx * Ui), where α₀ was set to zero in all scenarios. The outcome variable (Yi) was also generated from a Bernoulli distribution with py = expit(β₀ + β₁ * Xi + cy * Ui), with β₀ set to zero in all scenarios. This simulation process was repeated for each observation (i = 1 to n) and replicated across 8,000 datasets. The random numbers were generated using the standard random number generation functions available in the R software (version 4.2.1). and to ensure reproducibility, a fixed random seed was applied before each simulation run.

We considered four simulation scenarios to assess the performance of the Wald ratio methods. In scenarios A and B, the causal effect was set to zero (β₁ = 0), representing a null effect, while scenarios C and D involved a positive causal effect (β₁ = 1). Each scenario used a moderate sample size of 1,000 observations and varied the strength of the instrumental variable (α₁). The confounder effect was weak (0.01) in scenarios A and C, and strong (1.5) in scenarios B and D.

The bias was calculated as the difference between each of the three instrumental variable estimators (SEM, TSPS, and TSRI) and the corresponding Wald ratio estimates derived from logistic regression, LPM, and the transformation method [4, 7].

### Sensitivity analysis

Four scenarios were implemented for the sensitivity analysis: the first varied the sample size, the second adjusted the prevalence of the instrumental variable (IV), the third modified the prevalence of the exposure and outcome, and the fourth changed the confounder effect.

In the **first scenario**, the causal effect (β₁) was assigned values of 0 or 1, the confounder effects (cx and cy) were set at 0.01 or 1.5, and the sample size was varied across 50, 200, 500, 1000, and 1500.

In the **second scenario**, the causal effect (β₁) was set to 0 or 1, the confounder effects (cx and cy) were assigned values of 0.01 or 1.5, and the prevalence of the instrumental variable (pZ) was varied at 0.1, 0.2, 0.5, and 0.8.

In the **third scenario**, four distinct configurations were employed to vary the prevalence of the exposure and outcome (Supplementary Table 1). For each configuration, the causal effect (β₁) was assigned a value of 0 or 1, the confounder effects (cx and cy) were set at 0.01 or 1.5, and the instrument strength (α₁) varied across 0.01, 0.05, 0.1, 0.5, and 0.7. In the first configuration, α₀ = 0 and β₀ = 0; in the second, α₀ = 1 and β₀ = 1; in the third, α₀ = 3 and β₀ = 3; and in the fourth, α₀ = -2 and β₀ = -2. The prevalence of the exposure and outcome for each configuration is represented in Supplementary Table 1.

For the **fourth scenario**, the causal effect (β₁) was assigned a value of 0 or 1, and the confounder effects (cx and cy) varies across 0.01,0.1,0.5,1,1.5, and 2.

For the remaining parameters which specified in each scenario, the following values were assigned: instrument strength (α₁) = 0.01, sample size = 1000, prevalence of the instrumental variable (pZ) = 0.2, and α₀ = 0, β₀ = 0. The sensitivity analysis outputs were represented using box plot.

### Real data

In this real data analysis, we investigated the potential causal association between activity level (low or moderate) versus high and cancer in a MR framework using each existing method and the proposed method described above. For the risk factor of interest, we consider a dichotomized activity level. The dichotomous cancer status is used as the outcome of interest. Three dichotomous environmental confounders (treated_fertilizer, treated_insects, and treated_weeds) and sex were included in the analysis.

### Data source

The Golden Retriever Lifetime Study is a large, prospective cohort study established in 2012 to investigate risk factors for various health conditions in Golden dogs. Throughout each dog’s lifetime, owners and veterinarians complete an annual questionnaire, conduct clinical exams, and collect samples. All dogs in the study visit their veterinarian yearly for a physical exam and sample collection [12].

### Data management

#### Quality control of Single Nucleotide Polymorphisms (SNPs)

Genotyping data were provided as two datasets for the Affymetrix (ThermoFisher) Axiom Canine HD Array sets A and B, which needed to be merged into a final dataset by resolving replicate SNPs, known technical replicates, and gender conflicts. Quality control was performed by removing missing samples (--mind 0.05) and variants (--geno 0.05), excluding variants based on the Hardy–Weinberg equilibrium exact test (--hwe 1e-50 'midp'), applying a minor allele frequency threshold (--maf 0.01), and discarding samples with extremely low heterozygosity (< −3). First-degree relatives were removed (--king 0.177), and linkage disequilibrium (LD) pruning was conducted by removing pairwise SNPs with R^2^ > 0.8 using windows of 100 kb and a step size of 1 SNP [13, 14].

#### Phenotypes

Three phenotype files were downloaded: activity and lifestyle, neoplasia conditions, and environmental factors. From each file, the second-row records, representing diagnoses from baseline through the study year, were selected. The three phenotype files were then merged based on dog ID and study year. Finally, the data were filtered to retain only the last study year for each dog, indicating whether the dog had experienced cancer or not.

#### Phenotype identification

Cancer (outcome) was defined as 0 (no tumor) or 1 (at least one tumor diagnosed). Activity level (exposure) was categorized as 0 (mild, and moderate), or 1 (high). The variables for exposure to treated substances were defined as follows: sex (0:male, 1:female), any_treated_fertilizer (0: no, 1: yes), any_treated_insects (0: no, 1: yes), and any_treated_weeds (0: no, 1: yes) (confounders).

#### Associations test

Association tests for each individual SNP with activity level were conducted using Fisher's exact test when the assumptions of the chi-square test were violated; otherwise, the chi-square test was used. Five SNPs were chosen randomly from the top ten significant SNPs related to the exposure (activity level) were used to compare between the methods described above.

## Results

Across all scenarios, stronger instruments consistently led to better performance of the Wald ratio estimators, as reflected by the reduction in the interquartile range. The subsequent three paragraphs provide further detail.

Table 1 presents the bias of the Wald ratio using logistic regression across three estimation techniques (SEM, TSPS, and TSRI) under varying instrumental effect sizes. The median and interquartile range for the lowest instrumental effect compared to the highest effect were as follows: (1) For Scenario A: SEM: –0.04 (–1.04–0.97) vs. 0.00 (–0.17–0.17); TSPS: 0.10 (–2.92–3.09) vs. –0.01 (–0.51–0.48); and TSRI: 0.10 (–2.93–3.10) vs. –0.01 (–0.51–0.49). (2) For Scenario B: SEM: 0.00 (–1.01–1.04) vs. 0.00 (–0.21–0.21); TSPS: –0.16 (–1.89–1.52) vs. –0.04 (–0.67–0.60); and TSRI: –0.14 (–1.87–1.53) vs. –0.03 (–0.67–0.60). (3) For Scenario C: SEM: 0.77(−0.20–1.74) vs. 0.77(0.60–0.94); TSPS: 0.72(−2.24–3.61) vs. 0.77(0.26–1.25); and TSRI: 0.75(−2.47–3.87) vs. 0.77(0.22–1.29). (4) For Scenario D: SEM: 0.77(−0.28–1.83) vs 0.79(0.57–1.00); TSPS: 0.24(−1.64–1.98) vs. 0.69(0.05–1.33); and TSRI: 0.60(−1.28–2.37) vs. 0.78(0.09–1.45).

**Table 1:** Bias of the Wald ratio using logistic regression for estimating the causal effect, compared by three estimation technique (SEM, TSPS, TSRI) under varying instrumental effect sizes

Scenario*	Instrument strength (α_1_)	SEM Median (Q1–Q3)	TSPS Median (Q1–Q3)	TSRI Median (Q1–Q3)
A (β₁ = 0, cx = 0.01, cy = 0.01)	0.01	−0.04(−1.04–0.97)	0.10(−2.92–3.09)	0.10(−2.93–3.10)
	0.05	0.02(−0.91–0.97)	−0.10(−2.92–2.68)	−0.10(−2.92–2.68)
	0.1	−0.01(−0.85–0.85)	−0.01(−2.57–2.55)	−0.01(−2.57–2.55)
	0.5	0.00(−0.23–0.23)	−0.01(−0.68–0.68)	−0.01(−0.68–0.68)
	0.7	0.00(−0.17–0.17)	−0.01(−0.51–0.48)	−0.01(−0.51–0.49)
B (β₁ = 0, cx = 1.5, cy = 1.5)	0.01	0.00(−1.01–1.04)	−0.16(−1.89–1.52)	−0.14(−1.87–1.53)
	0.05	−0.03(−1.01–1.01)	−0.15(−1.90–1.56)	−0.13(−1.89–1.57)
	0.1	−0.01(−0.89–0.90)	−0.11(−1.81–1.48)	−0.10(−1.79–1.49)
	0.5	0.01(−0.27–0.29)	−0.07(−0.89–0.74)	−0.07(−0.88–0.75)
	0.7	0.00(−0.21–0.21)	−0.04(−0.67–0.60)	−0.03(−0.67–0.60)
C (β₁ = 1, cx = 0.01, cy = 0.01)	0.01	0.77(−0.20–1.74)	0.72(−2.24–3.61)	0.75(−2.47–3.87)
	0.05	0.79(−0.18–1.72)	0.66(−2.19–3.55)	0.68(−2.40–3.80)
	0.1	0.73(−0.09–1.59)	0.83(−1.74–3.30)	0.85(−1.92–3.53)
	0.5	0.76(0.53–0.99)	0.73(0.06–1.45)	0.74(0.01–1.51)
	0.7	0.77(0.60–0.94)	0.77(0.26–1.25)	0.77(0.22–1.29)
D (β₁ = 1, cx = 1.5, cy = 1.5)	0.01	0.77(−0.28–1.83)	0.24(−1.64–1.98)	0.60(−1.28–2.37)
	0.05	0.78(−0.22–1.80)	0.34(−1.55–2.05)	0.70(−1.22–2.47)
	0.1	0.77(−0.12–1.68)	0.26(−1.50–1.93)	0.59(−1.20–2.29)
	0.5	0.79(0.50–1.08)	0.57(−0.30–1.43)	0.72(−0.17–1.63)
	0.7	0.79(0.57–1.00)	0.69(0.05–1.33)	0.78(0.09–1.45)

*The prevalence of the exposure and outcome for each row is represented in Supplementary Table 1, scenario 1

Table 2 presents the bias of the Wald ratio using LPM across three estimation techniques (SEM, TSPS, and TSRI) under varying instrumental effect sizes. The median and interquartile range for the lowest instrumental effect compared to the highest effect were as follows: (1) For Scenario A: SEM: − 0.15(−4.14–3.84) vs 0.01(− 0.63–0.66); TSPS: 0.00(− 0.01–0.01) vs. 0.00(− 0.01–0.00); and TSRI: 0.00(− 0.02–0.02) vs. 0.00(− 0.01–0.00). (2) For Scenario B: SEM: − 0.09(−4.11– 4.05) vs. 0.02(− 0.75–0.78); TSPS: − 0.23(− 4.04– 3.50) vs. − 0.04(− 0.48– 0.41); and TSRI: − 0.23(− 4.01–3.51) vs. − 0.04(− 0.48–0.41). (3) For Scenario C: SEM: 0.03(− 3.78–3.95) vs. 0.01(− 0.59–0.67); TSPS: 0.00(− 0.04–0.05) vs. 0.02(0.00–0.04); and TSRI: 0.02(− 0.21–0.26) vs. 0.02(− 0.03–0.08). (4) For Scenario D: SEM: 0.08(− 3.81–4.03) vs 0.19(− 0.54–0.91); TSPS: − 0.39(− 4.12–3.00) vs. 0.09(− 0.34–0.53); and TSRI: − 0.09(− 3.75–3.39) vs. 0.16(− 0.28–0.63).

**Table 2:** Bias of the Wald ratio using LPM for estimating the causal effect, compared by three estimation technique (SEM, TSPS, TSRI) under varying instrumental effect sizes

Scenario*	Instrument strength (α_1_)	SEM Median (Q1–Q3)	TSPS Median (Q1–Q3)	TSRI Median (Q1–Q3)
A (β₁ = 0, cx = 0.01, cy = 0.01)	0.01	−0.15(−4.14–3.84)	0.00(−0.01–0.01)	0.00(−0.02–0.02)
	0.05	0.11(−3.59–3.85)	0.00(−0.01–0.01)	0.00(−0.02–0.02)
	0.1	0.01(−3.39–3.39)	0.00(−0.01–0.01)	0.00(−0.02–0.02)
	0.5	0.00(−0.89–0.89)	0.00(−0.01–0.01)	0.00(−0.01–0.01)
	0.7	0.01(−0.63–0.66)	0.00(−0.01–0.00)	0.00(−0.01–0.00)
B (β₁ = 0, cx = 1.5, cy = 1.5)	0.01	−0.09(−4.11–4.05)	−0.23(−4.04–3.50)	−0.23(−4.01–3.51)
	0.05	−0.15(−3.98–3.81)	−0.26(−3.84–3.38)	−0.26(−3.84–3.38)
	0.1	−0.03(−3.57–3.59)	−0.14(−3.44–3.10)	−0.14(−3.40–3.10)
	0.5	0.03(−1.05–1.08)	−0.07(−0.76–0.63)	−0.07(−0.76–0.63)
	0.7	0.02(−0.75–0.78)	−0.04(−0.48–0.41)	−0.04(−0.48–0.41)
C (β₁ = 1, cx = 0.01, cy = 0.01)	0.01	0.03(−3.78–3.95)	0.00(−0.04–0.05)	0.02(−0.21–0.26)
	0.05	0.10(−3.72–3.91)	0.01(−0.03–0.06)	0.02(−0.21–0.27)
	0.1	−0.11(−3.31–3.32)	0.01(−0.01–0.07)	0.03(−0.17–0.27)
	0.5	0.04(−0.86–0.93)	0.02(0.00–0.04)	0.02(−0.04–0.10)
	0.7	0.01(−0.59–0.67)	0.02(0.00–0.04)	0.02(−0.03–0.08)
D (β₁ = 1, cx = 1.5, cy = 1.5)	0.01	0.08(−3.81–4.03)	−0.39(−4.12–3.00)	−0.09(−3.75–3.39)
	0.05	0.09(−3.58–3.98)	−0.36(−3.87–3.14)	−0.03(−3.47–3.51)
	0.1	0.11(−3.27–3.54)	−0.33(−3.68–2.63)	−0.01(−3.32–2.99)
	0.5	0.21(−0.81–1.21)	−0.01(−0.69–0.66)	0.11(−0.55–0.82)
	0.7	0.19(−0.54–0.91)	0.09(−0.34–0.53)	0.16(−0.28–0.63)

*The prevalence of the exposure and outcome for each row is represented in Supplementary Table 1, scenario 1

Table 3 presents the bias of the Wald ratio using transformation across three estimation techniques (SEM, TSPS, and TSRI) under varying instrumental effect sizes. The median and interquartile range for the lowest instrumental effect compared to the highest effect were as follows: (1) For Scenario A: SEM: − 0.15(− 4.14–3.84) vs 0.01(− 0.61–0.63); TSPS: 0.00(− 0.01–0.01) vs. 0.00(− 0.02–0.02); and TSRI: 0.00(− 0.01–0.01) vs 0.00(− 0.02–0.02). (2) For Scenario B: SEM: 0.00(− 4.09–4.12) vs. 0.02(− 0.73–0.76); TSPS: − 0.20(− 3.92–3.43) vs. − 0.05(− 0.47–0.38); and TSRI: − 0.19(− 3.90–3.44) vs. − 0.05(− 0.46–0.38). (3) For Scenario C: SEM: 0.03(− 3.79–3.94) vs. 0.04(− 0.58–0.68); TSPS: 0.00(− 0.01–0.01) vs. 0.03(0.01–0.06); and TSRI: 0.02(− 0.22–0.26) vs. 0.04(− 0.02–0.10). (4) For Scenario D: SEM: 0.05(− 4.06–4.37) vs 0.13(− 0.64–0.90); TSPS: − 0.40(−4.38–3.19) vs. 0.02(− 0.39–0.44); and TSRI: −0.08(− 3.97–3.57) vs. 0.09(− 0.32–0.53).

**Table 3:** Bias of the Wald ratio using transformation for estimating the causal effect, compared by three estimation technique (SEM, TSPS, TSRI) under varying instrumental effect sizes

Scenario*	Instrument strength (${\alpha }_{1}$)	SEM Median (Q1–Q3)	TSPS Median (Q1–Q3)	TSRI Median (Q1–Q3)
A (β₁ = 0, cx = 0.01, cy = 0.01)	0.01	−0.15(−4.14–3.84)	0.00(−0.01–0.01)	0.00(−0.01–0.01)
	0.05	0.11(−3.59–3.85)	0.00(−0.01–0.01)	0.00(−0.01–0.01)
	0.1	0.01(−3.39–3.38)	0.00(−0.01–0.01)	0.00(−0.01–0.01)
	0.5	0.00(−0.87–0.87)	0.00(−0.02–0.02)	0.00(−0.02–0.02)
	0.7	0.01(−0.61–0.63)	0.00(−0.02–0.02)	0.00(−0.02–0.02)
B (β₁ = 0, cx = 1.5, cy = 1.5)	0.01	0.00(−4.09–4.12)	−0.20(−3.92–3.43)	−0.19(−3.90–3.44)
	0.05	−0.15(−4.01–3.97)	−0.21(−3.82–3.40)	−0.21(−3.81–3.42)
	0.1	0.01(−3.52–3.57)	−0.12(−3.40–3.07)	−0.11(−3.38–3.10)
	0.5	0.03(−1.02–1.08)	−0.07(−0.74–0.60)	−0.07(−0.74–0.61)
	0.7	0.02(−0.73–0.76)	−0.05(−0.47–0.38)	−0.05(−0.46–0.38)
C (β₁ = 1, cx = 0.01, cy = 0.01)	0.01	0.03(−3.79–3.94)	0.00(−0.01–0.01)	0.02(−0.22–0.26)
	0.05	0.10(−3.76–3.90)	0.00(−0.01–0.01)	0.02(−0.22–0.25)
	0.1	−0.11(−3.36–3.30)	0.00(−0.01–0.01)	0.03(−0.18–0.24)
	0.5	0.05(−0.86–0.93)	0.02(0.00–0.04)	0.03(−0.04–0.10)
	0.7	0.04(−0.58–0.68)	0.03(0.01–0.06)	0.04(−0.02–0.10)
D (β₁ = 1, cx = 1.5, cy = 1.5)	0.01	0.05(−4.06–4.37)	−0.40(−4.38–3.19)	−0.08(−3.97–3.57)
	0.05	0.10(−3.85–4.12)	−0.39(−4.06–3.27)	−0.06(−3.66–3.65)
	0.1	0.11(−3.42–3.74)	−0.32(−3.74–2.85)	−0.04(−3.41–3.22)
	0.5	0.15(−0.91–1.21)	−0.06(−0.76–0.59)	0.05(−0.62–0.74)
	0.7	0.13(−0.64–0.90)	0.02(−0.39–0.44)	0.09(−0.32–0.53)

*The prevalence of the exposure and outcome for each row is represented in Supplementary Table 1, scenario 1

### Sensitivity analysis

Supplementary Fig. 1 illustrates the influence of increasing sample size on the performance of the Wald ratio using logistic regression, LPM, and the transformation approach in estimating causal effects under SEM, TSPS, and TSRI Frameworks. For the SEM approach, a consistent bias was observed across all four scenarios. In contrast, for TSPS and TSRI, increasing the sample size led to a reduction in bias when the confounder effect was low, whereas under high confounding, the Wald ratio estimated using LPM and the transformation method showed less improvement. Conversely, the Wald method based on logistic regression exhibited a reduction in bias across all scenarios as the sample size increased; however, the overall bias remained high.

Supplementary Fig. 2 illustrates the effect of increasing the proportion of instrumental variables on the performance of the Wald ratio—using logistic regression, LPM, and the transformation approach—in estimating causal effects within the SEM, TSPS, and TSRI frameworks. The results showed that the bias remained relatively consistent across all scenarios as the proportion of instrumental variables increased.

Supplementary Figs. 3, 4, 5, and 6 illustrate the effect of varying the prevalence of the exposure and outcome on the performance of the Wald ratio—applied through logistic regression, LPM, and the transformation approach—in estimating causal effects within the SEM, TSPS, and TSRI frameworks. The findings indicated that increasing the strength of the instrumental variable led to a reduction in bias, with the best performance observed at instrumental strengths of 0.5 and 0.7. Additionally, the LPM and transformation methods demonstrated comparatively lower bias in the TSPS and TSRI models, particularly when the confounder effect was low. However, increasing the prevalence of the exposure and outcome resulted in higher bias. It was noted that when the prevalence of the exposure and outcome ranged from 0.67 to 0.84 and the instrumental strength was high (0.7), the bias of the LPM and transformation methods was slightly higher but comparable to that observed when the prevalence ranged from 0.5 to 0.62. However, when the prevalence of the exposure and outcome ranged from 0.12 to 0.23, the bias was higher to that observed when the prevalence ranged from 0.67 to 0.84.

Supplementary Fig. 7 presents the impact of changing the confounder effect on both the exposure and the outcome on the performance of the Wald ratio—implemented using logistic regression, LPM, and the transformation approach—in estimating causal effects within the SEM, TSPS, and TSRI frameworks. The results showed that the LPM and transformation approaches produced relatively lower bias in the TSPS framework when the confounder effect was below 0.1, under both the null and positive causal effect scenarios. Conversely, in the TSRI framework, these approaches exhibited considerably higher bias under the positive causal effect scenario, while the bias remained low under the null causal effect.

### Real data analysis

Table 4 presents the characteristics of the five SNPs analyzed in the real data analysis using SEM approach. The estimated causal effect (β₁) ranged from 0.05 to 0.09 and the instrument strength (α₁) was approximately 0.7. Additionally, two confounders exhibited strong effects.

**Table 4:** The characteristics of the five SNPs in real data analysis using SEM method

Parameters	Affx-205724246_A	Affx-205954640_C	Affx-206537074_C	Affx-206467463_G	Affx-206333877_T
α₁	0.67 (0.51–0.83)	0.64 (0.49–0.79)	0.67 (0.51–0.84)	0.67 (0.51–0.83)	0.69 (0.52–0.84)
Cx_1_	− 0.26 ( − 0.48– − 0.04)	− 0.27 ( − 0.49– − 0.05)	− 0.28 ( − 0.50– − 0.06)	− 0.27 ( − 0.49– − 0.05)	− 0.27 ( − 0.48– − 0.04)
Cx_2_	− 0.11 ( − 0.36–0.13)	− 0.11 ( − 0.36–0.13)	− 0.14 ( − 0.38–0.11)	− 0.12 ( − 0.36–0.13)	− 0.11 ( − 0.36–0.13)
Cx_3_	− 0.64 ( − 0.96– − 0.33)	− 0.66 ( − 0.98– − 0.34)	− 0.64 ( − 0.96– − 0.32)	− 0.66 ( − 0.98– − 0.35)	− 0.66 ( − 0.97– − 0.34)
Cx_4_	− 0.23 ( − 0.55–0.10)	− 0.21 ( − 0.53–0.11)	− 0.21 ( − 0.53–0.12)	− 0.20 ( − 0.53–0.12)	− 0.21 ( − 0.54–0.11)
β₁	0.09 ( − 0.30–0.48)	0.08 ( − 0.31–0.47)	0.08 ( − 0.30–0.47)	0.08 ( − 0.31–0.47)	0.05 ( − 0.34–0.44)
Cy_1_	− 0.19 ( − 0.48–0.09)	− 0.18 ( − 0.47–0.10)	− 0.22 ( − 0.51–0.07)	− 0.19 ( − 0.48–0.09)	− 0.19 ( − 0.47–0.10)
Cy_2_	0.07 ( − 0.26–0.41)	0.07 ( − 0.26–0.40)	0.06 ( − 0.28–0.39)	0.08 ( − 0.25–0.41)	0.09 ( − 0.24–0.42)
Cy_3_	0.84 (0.35–1.32)	0.85 (0.37–1.33)	0.77 (0.29–1.26)	0.85 (0.37–1.33)	0.87 (0.39–1.35)
Cy_4_	0.40 ( − 0.05–0.84)	0.34 ( − 0.09–0.78)	0.44 ( − 0.01–0.89)	0.35 ( − 0.09–0.78)	0.34 ( − 0.10–0.78)
Prevalence of x	0.18	0.18	0.18	0.18	0.18
Prevalence of y	0.091	0.091	0.091	0.091	0.091
Sample size	2337	2339	2323	2340	2336

Cx1 sex; Cx2: any_treated_insects; Cx3: any_treated_fertilizer; Cx4: any_treated_weeds

The valuepresented is measured on the log-odds scale (95% CI)

Table 5 shows the causal effects of activity level on cancer using data from the Golden Retriever Lifetime Study. The bias of the Wald ratio using LPM and transformation was much higher than that of the Wald ratio using logistic regression under SEM, whereas the bias of the Wald ratio using LPM and transformation was much lower than that of logistic regression under TSPS and TSRI. For example, for the SNP Affx-205724246_A: under SEM, the bias for logistic regression was –0.14, for LPM it was –1.30, and for the transformation method it was –1.45. Under TSPS and TSRI, the bias for logistic regression was 1.24, for LPM 0.08, and for the transformation method –0.07.

**Table 5:** Causal estimates of activity level on cancer from the Golden Retriever Lifetime Study, using three different methods (SEM, TSPS, TSRI) compared with the Wald ratio methods

SNP	Method	Causal effect	Wald ratio		
			Logistic regression (bias)	LPM (log odds scale) (bias)	Transformation (log odds scale) (bias)
Affx-205724246_A	SEM	0.09	0.23 ( − 0.14)	1.39 ( − 1.3)	1.54 ( − 1.45)
	TSPS	1.47	0.23 (1.24)	1.39 (0.08)	1.54 ( − 0.07)
	TSRI	1.47	0.23 (1.24)	1.39 (0.08)	1.54 ( − 0.07)
Affx-205954640_C	SEM	0.08	0.18 ( − 0.1)	1.16 ( − 1.08)	1.23 ( − 1.15)
	TSPS	0.98	0.18 (0.8)	1.16 ( − 0.18)	1.23 ( − 0.25)
	TSRI	0.98	0.18 (0.8)	1.16 ( − 0.18)	1.23 ( − 0.25)
Affx-206537074_C	SEM	0.08	0.22 ( − 0.14)	1.38 ( − 1.3)	1.49 ( − 1.41)
	TSPS	1.27	0.22 (1.05)	1.38 ( − 0.11)	1.49 ( − 0.22)
	TSRI	1.27	0.22 (1.05)	1.38 ( − 0.11)	1.49 ( − 0.22)
Affx-206467463_G	SEM	0.08	0.19 ( − 0.11)	1.22 ( − 1.14)	1.31 ( − 1.23)
	TSPS	1.02	0.19 (0.83)	1.22 ( − 0.2)	1.31 ( − 0.29)
	TSRI	1.02	0.19 (0.83)	1.22 ( − 0.2)	1.31 ( − 0.29)
Affx-206333877_T	SEM	0.05	0.2 ( − 0.15)	1.28 ( − 1.23)	1.37 ( − 1.32)
	TSPS	0.94	0.2 (0.74)	1.28 ( − 0.34)	1.37 ( − 0.43)
	TSRI	0.94	0.2 (0.74)	1.28 ( − 0.34)	1.37 ( − 0.43)

## Discussion

In Mendelian Randomization studies, when calculating the causal estimate using the Wald ratio and both the exposure and outcome are continuous, the estimate reflects the change in the outcome per unit change in the exposure. When the exposure is binary, genetic associations with the exposure are typically estimated using logistic regression, resulting in log odds ratios. Consequently, the causal estimate represents the change in the outcome per unit change in the log odds of the exposure. When the outcome is binary, the causal estimate reflects the change in the log odds of the outcome (i.e., the log odds ratio for the disease, in the case of logistic regression) per unit increase in the exposure due to the genetic instrument. When both the exposure and outcome are binary, the causal estimate represents the change in the log odds of the outcome per unit change in the log odds of the exposure.

The Wald ratio using logistic regression does not provide the true causal effect when both the exposure and outcome are binary, due to the non-collapsibility of the odds ratio. To approximate the true causal effect, one approach is to first transform the logistic regression coefficients to the linear probability scale, conduct the Wald ratio calculation, and then transform the estimate back to the log odds scale. Therefore, this article presents the biases of the Wald ratio using logistic regression, LPM, and transformation methods for a binary risk factor and outcome, compared across SEM, TSPS, and TSRI estimation techniques.

In Structural Equation Modeling (SEM), each dependent variable has its own equation, and unlike two-stage regression, these equations are estimated simultaneously rather than sequentially (two- stage regression). When both the exposure and outcome are continuous, SEM can produce a causal effect equivalent to two-stage regression, but only if the correlation between the error term (from regressing x on the IV) and the error term (from regressing y on x) is incorporated into the SEM model. However, when both the exposure and outcome are binary, specifying such an error correlation is not feasible as there is no error term in logistic regression. Consequently, the causal effect in the binary SEM setting was estimated using logistic regression that included the exposure and outcome-related covariates, without accounting for the influence of the instrumental variable. Furthermore, the exposure scale in the SEM model differs from that in the TSPS model, whereas in the TSRI model, it shares the same scale as SEM but incorporates an additional residual term. Consequently, the causal effect estimates derived from SEM and two-stage methods are expected to differ. In our applied analysis, SNP Affx-205724246_A yielded an effect estimate of 0.09 in SEM compared with 1.09 under TSPS/TSRI, reflecting these methodological distinctions. From the results, the bias of the Wald ratio using logistic regression was smaller under the null causal effect than under the positive causal effect when using the SEM estimation method. In addition, the bias of Wald ratio using logistic regression under SEM in case of high confounder effect and low instrumental was much lower than that of LPM and Transformation method. Several authors have recommended that Mendelian Randomization studies should focus on testing the causal null hypothesis rather than estimating the causal effect [15]. Therefore, the Wald ratio using logistic regression may be useful for testing whether the causal effect is null or not.

In the current analysis, we reported the absolute bias (the difference between the true value and the estimator) rather than the percentage bias, since percentage bias can be misleading due to its dependence on the scale of the true parameter. As previously discussed, the scale of the causal effect in the SEM model differs from that in the TSPS and TSRI approaches. Consequently, using percentage bias would not allow for a valid comparison across these different modelling frameworks.

Based on the characteristics of the real data, the expected first (Q1) and third (Q3) quartiles of the bias for each method were obtained from the scenario with no causal effect, high confounding, and α₁ = 0.7 (Supplementary Fig. 6. For the SEM method, the expected bias of the Wald ratio using logistic regression, LPM, and the transformation approach were (− 0.26–0.27) (− 1.36–1.3), and (− 1.39–1.37), respectively. For the TSPS method, the corresponding ranges were (− 0.74–0.68), (− 1.03–0.88), and (− 1.02–0.91), respectively. For the TSRI method, the ranges were (− 0.74–0.69), (− 1.02–0.88), and (− 1.01–0.92), respectively. These results indicate that the Wald ratio using logistic regression under the SEM framework produced the smallest bias, which was consistent with the findings from the real data analysis (Table 5). Thus, applying the Wald ratio with logistic regression in our real data analysis to estimate the causal effect yields an estimate without accounting for the instrumental variable but has bias lower than estimates obtained from the LPM or the transformation approach. Although all SNPs exhibit similar effects on x (Table 4), their predicted outcome probabilities differ significantly, leading to variation in the estimated causal effect of x on y for the TSPS and TSRI methods (Table 5). This variability is reflected in the Wald ratio bias under logistic regression, where the bias for two SNPs in the TSPS and TSRI frameworks was notably larger than anticipated. It is evident that the causal effect estimates derived from the SEM were relatively consistent across the five SNPs, as the SEM model does not incorporate the instrumental variable. The small differences observed were due to the varying values of missing data across SNPs. This enabled us to compare the SEM estimates with those from the TSPS and TSRI models using the same sample size.

The simulation study recommended the following:

1. The results of Wald ratio using LPM is comparable to Wald ratio using Transformation.
2. If the instrument strength is weak (< 0.5) and the confounder effect exceeds 0.1, the Wald ratio estimated using logistic regression produces lower bias under the SEM approach.
3. If the confounder effect less than 0.1 and the prevalence of the outcome or the exposure is within 0.5 to 0.65, the Wald ratio using LPM produces lower bias under TSPS approach. However, if coefficient of LPM wasn’t available, Wald ratio using transformation method can be used as it gives comparable bias with Wald ratio using LPM.
4. If the outcome or exposure prevalence is approximately 0.85 and the instrument strength is high (0.7), the Wald ratio using LPM under the TSPS approach shows slightly higher bias compared to when the prevalence is within 0.5–0.65 with the same level of instrument strength.
5. When the prevalence of the outcome or exposure is low (0.12 to 0.23), the resulting bias is greater compared to when the prevalence is high (0.67 to 0.84).
6. If the instrument strength is high (0.5 or 0.7), the confounder effect exceed 0.1 and under null causal effect, the Wald ratio using logistic regression under SEM approach has lower bias compared to Wald ratio using LPM under TSPS approach.
7. If the instrument strength is high (0.5 or 0.7), and the confounder effect exceed 0.1 and under positive causal effect, the Wald ratio using LPM under TSPS approach has lower bias compared to Wald ratio using logistic regression under SEM approach.
8. The bias of the Wald ratio using LPM is greater under the TSRI approach than under the TSPS approach when the causal effect is positive.

## Supplementary Information

Below is the link to the electronic supplementary material.

Supplementary Material 1

## Data Availability

Data from the Golden Retriever Lifetime Study were used in this analysis, including SNP genotype data, Conditions–Neoplasia data, Environment, Exposures and Living Conditions data, and Activity and Lifestyle data. All datasets are publicly accessible at: https://datacommons.morrisanimalfoundation.org/datasets.
