# Supplementary material for: Transformation to estimate the causal effect in Mendelian randomization study with binary risk factor and outcome
Source: BMC Bioinformatics. 2026 Feb 27;27:72. doi: 10.1186/s12859-026-06388-1 (PMC13023191; doi:10.1186/s12859-026-06388-1)
Supplement: Supplementary file 1 — Supplementary Material 1 [file 12859_2026_6388_MOESM1_ESM.docx]

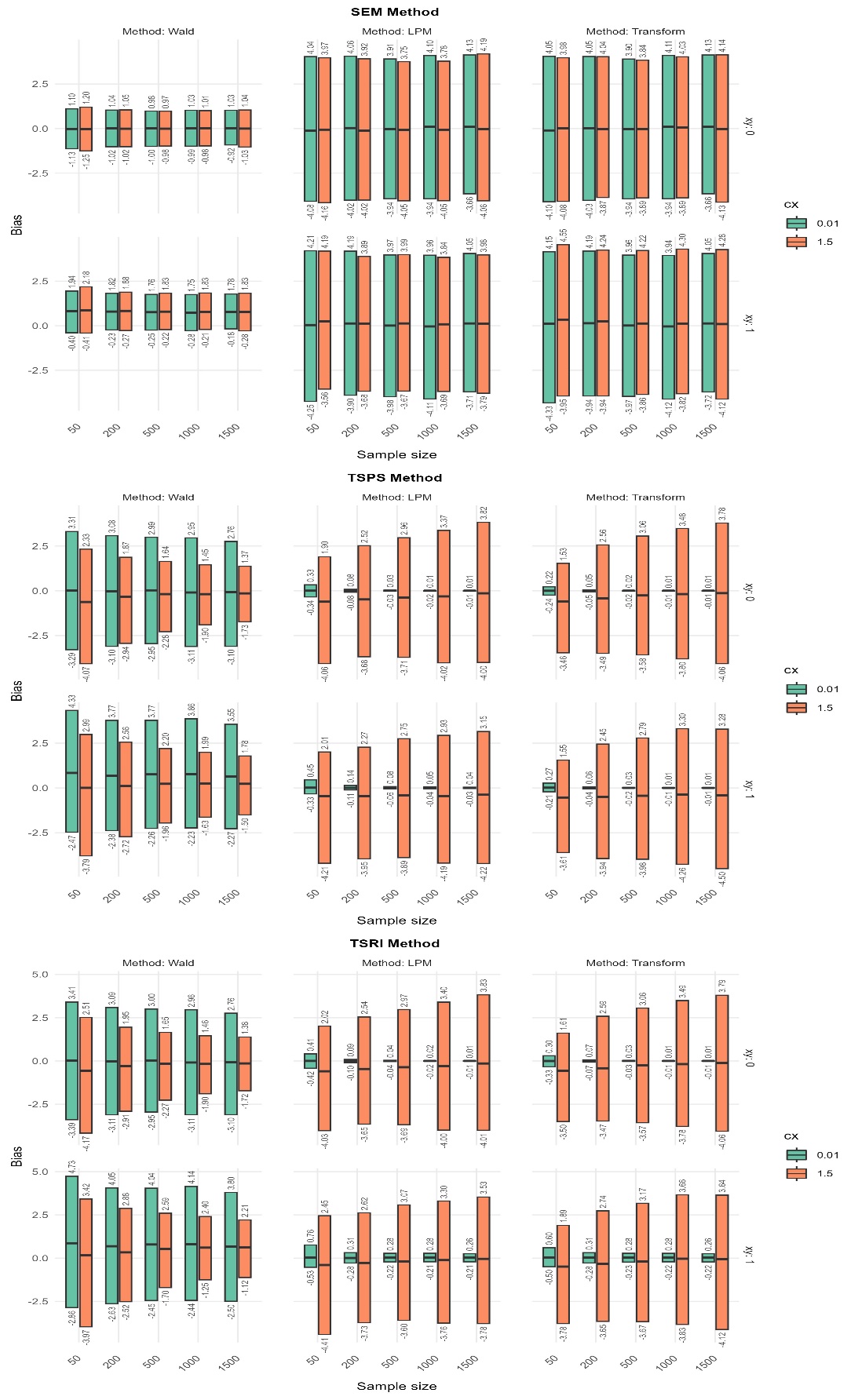


Figure 1: Variation in sample size with confounder effects between exposure (cx) and outcome (cy) set at 0.01 or 1.5, and the causal effect between exposure and outcome (β₁=xy) set at 0 or 1.

Wald: Wald ratio using logistic regression
LPM: Wald ratio using linear probability model
Transform: Wald ratio using Transformation.


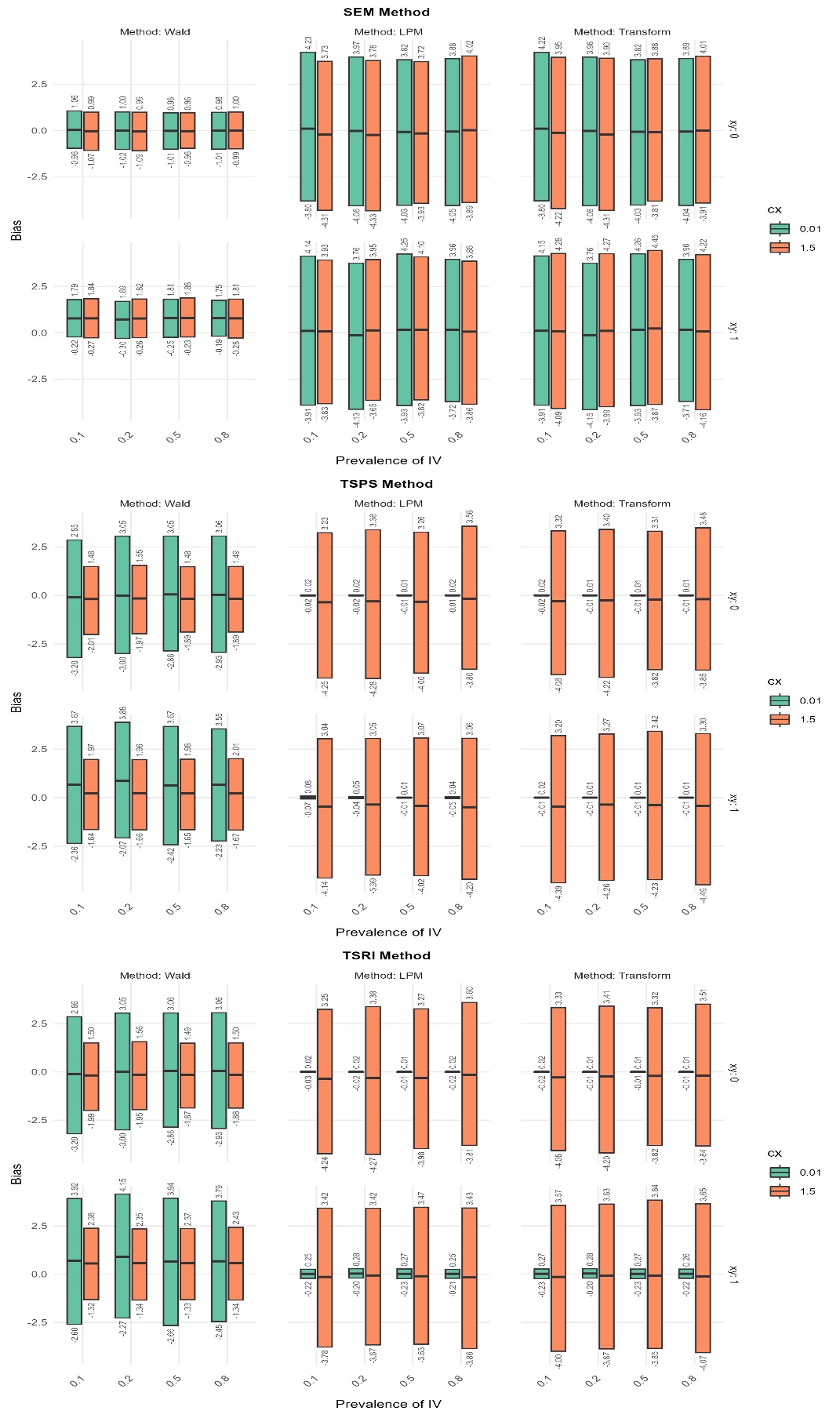


Figure 2: Variation in SNP prevalence with confounder effects between exposure (cx) and outcome (cy) set at 0.01 or 1.5, and the causal effect between exposure and outcome (β₁=xy) set at 0 or 1.

Wald: Wald ratio using logistic regression
LPM: Wald ratio using linear probability model
Transform: Wald ratio using Transformation.


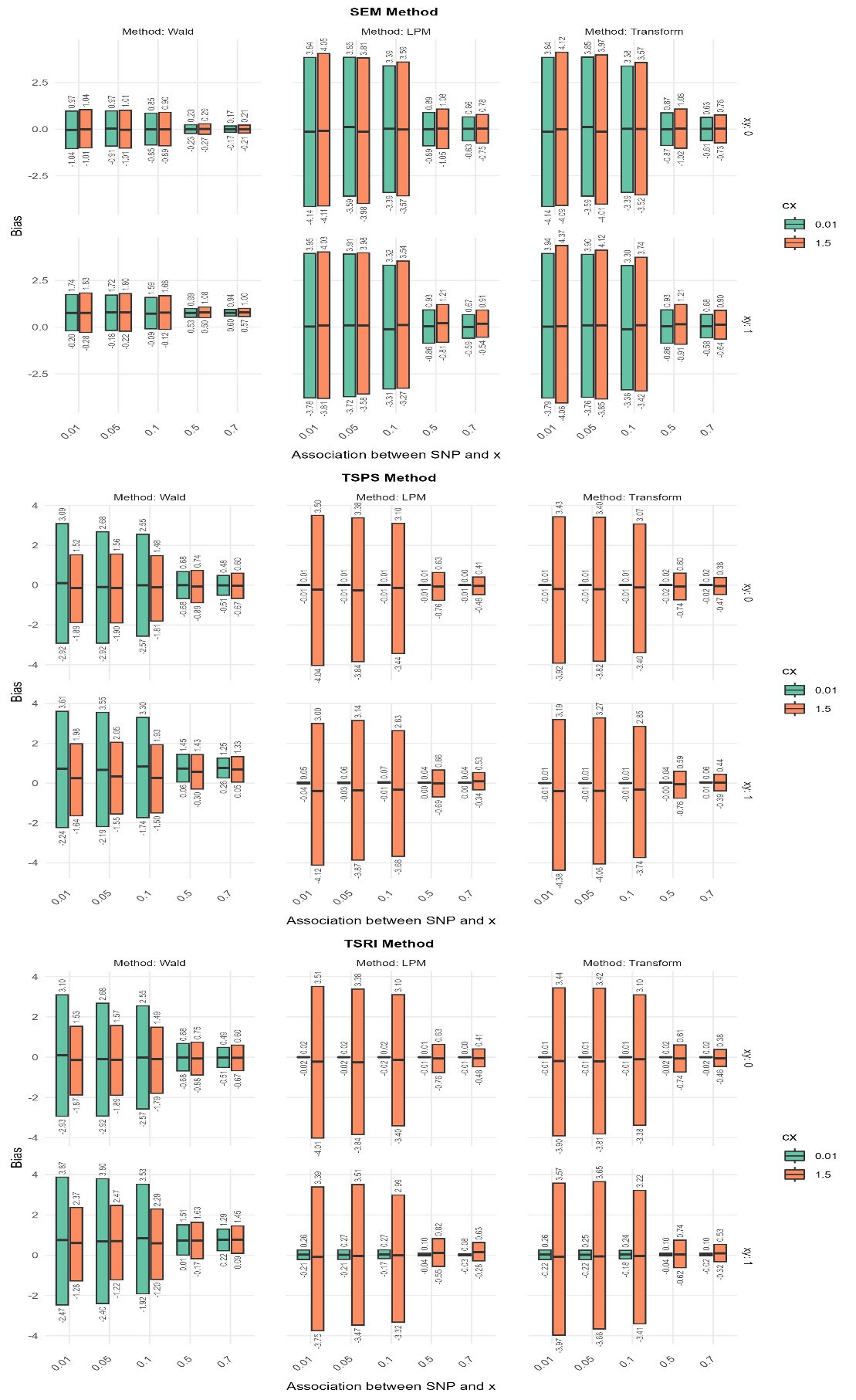
Figure 3: Variation in Instrument Strength with confounder effects between exposure (cx) and outcome (cy) set at 0.01 or 1.5, and the causal effect between exposure and outcome (β₁=xy) set at 0 or 1 α₀ = 0, β₀=0. Prevalence of exposure and outcome presented in table 1.

Wald: Wald ratio using logistic regression
LPM: Wald ratio using linear probability model
Transform: Wald ratio using Transformation.


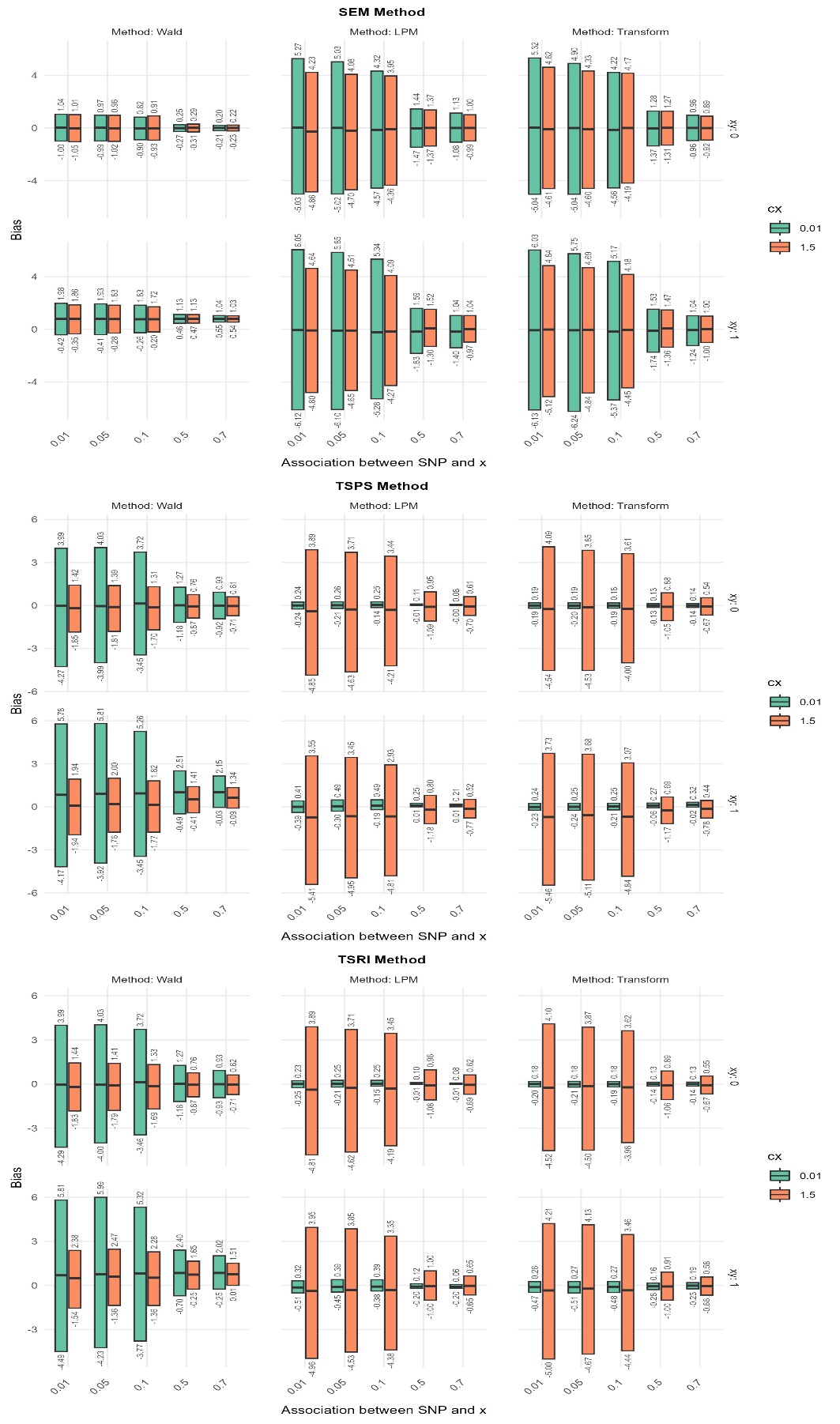


Figure 4: Variation in Instrument Strength with confounder effects between exposure (cx) and outcome (cy) set at 0.01 or 1.5, and the causal effect between exposure and outcome (β₁=xy) set at 0 or 1, α₀ = 1, β₀=1. Prevalence of exposure and outcome presented in table 1

Wald: Wald ratio using logistic regression
LPM: Wald ratio using linear probability model
Transform: Wald ratio using Transformation.


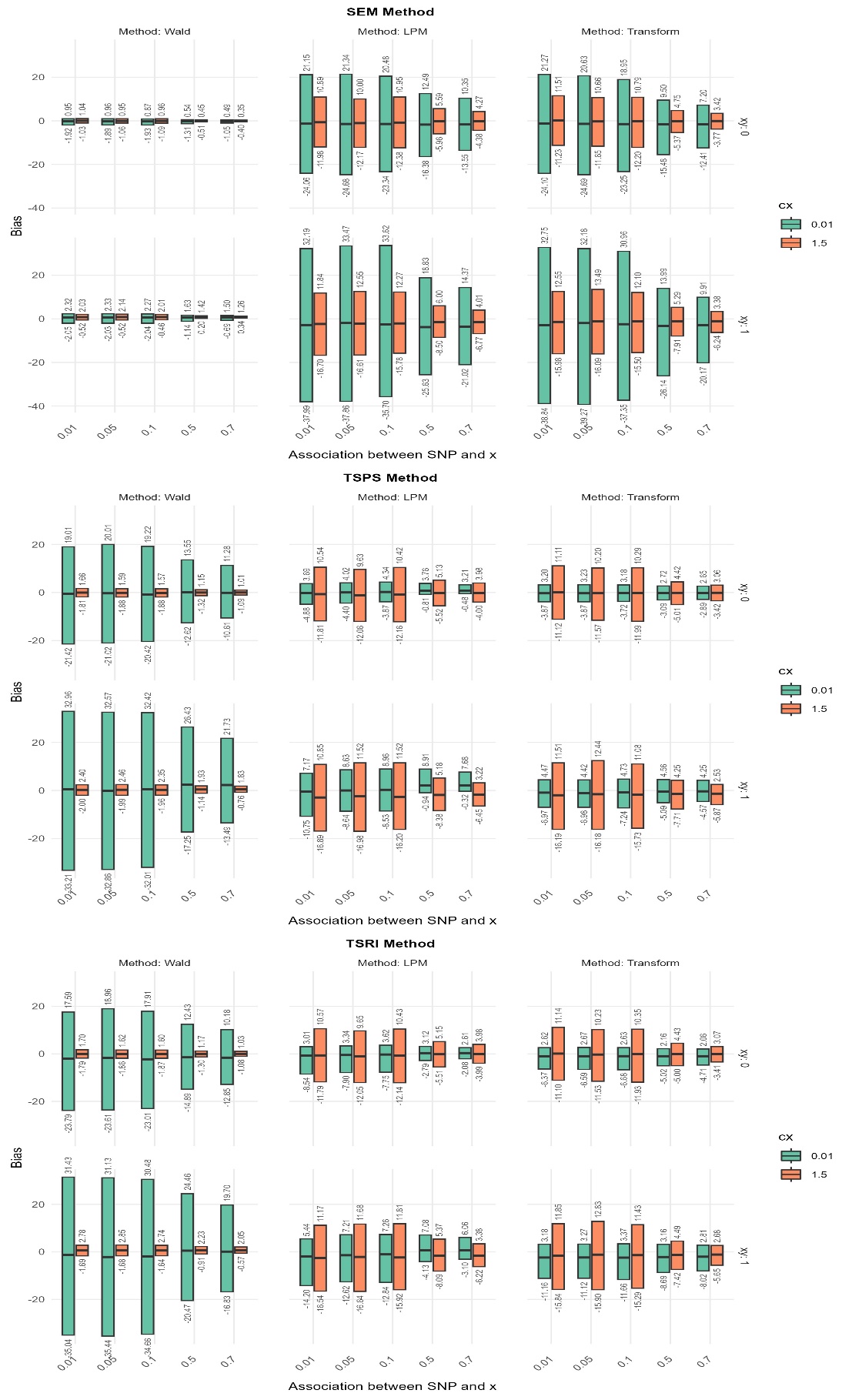


Figure 5: Variation in Instrument Strength with confounder effects between exposure (cx) and outcome (cy) set at 0.01 or 1.5, and the causal effect between exposure and outcome (β₁=xy) set at 0 or 1, α₀ = 3, β₀=3. Prevalence of exposure and outcome presented in table 1.

Wald: Wald ratio using logistic regression
LPM: Wald ratio using linear probability model
Transform: Wald ratio using Transformation.


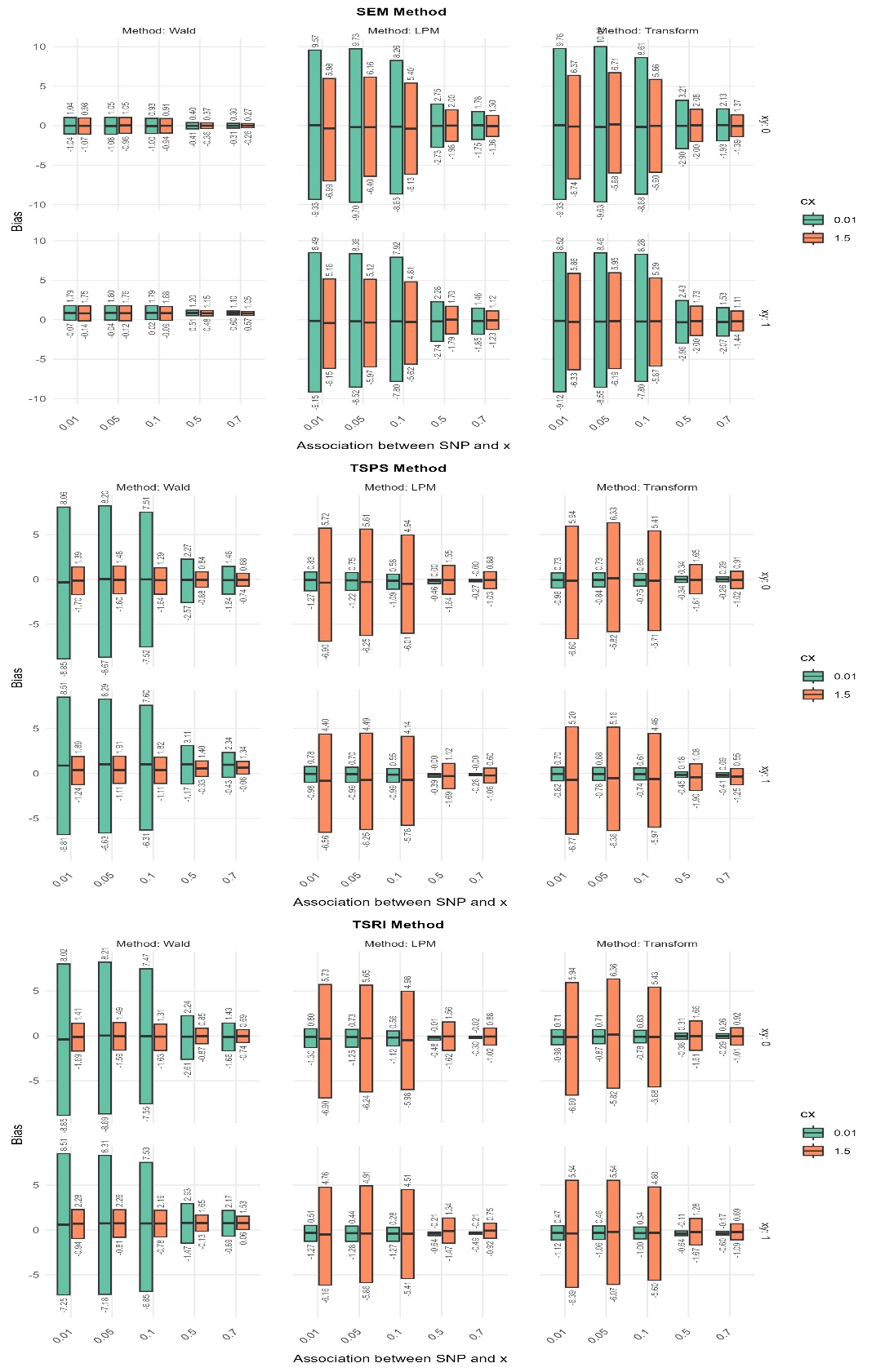


Figure 6: Variation in Instrument Strength with confounder effects between exposure (cx) and outcome (cy) set at 0.01 or 1.5, and the causal effect between exposure and outcome (β₁=xy) set at 0 or 1, α₀ = -2, β₀=-2. Prevalence of exposure and outcome presented in table 1.

Wald: Wald ratio using logistic regression
LPM: Wald ratio using linear probability model
Transform: Wald ratio using Transformation.


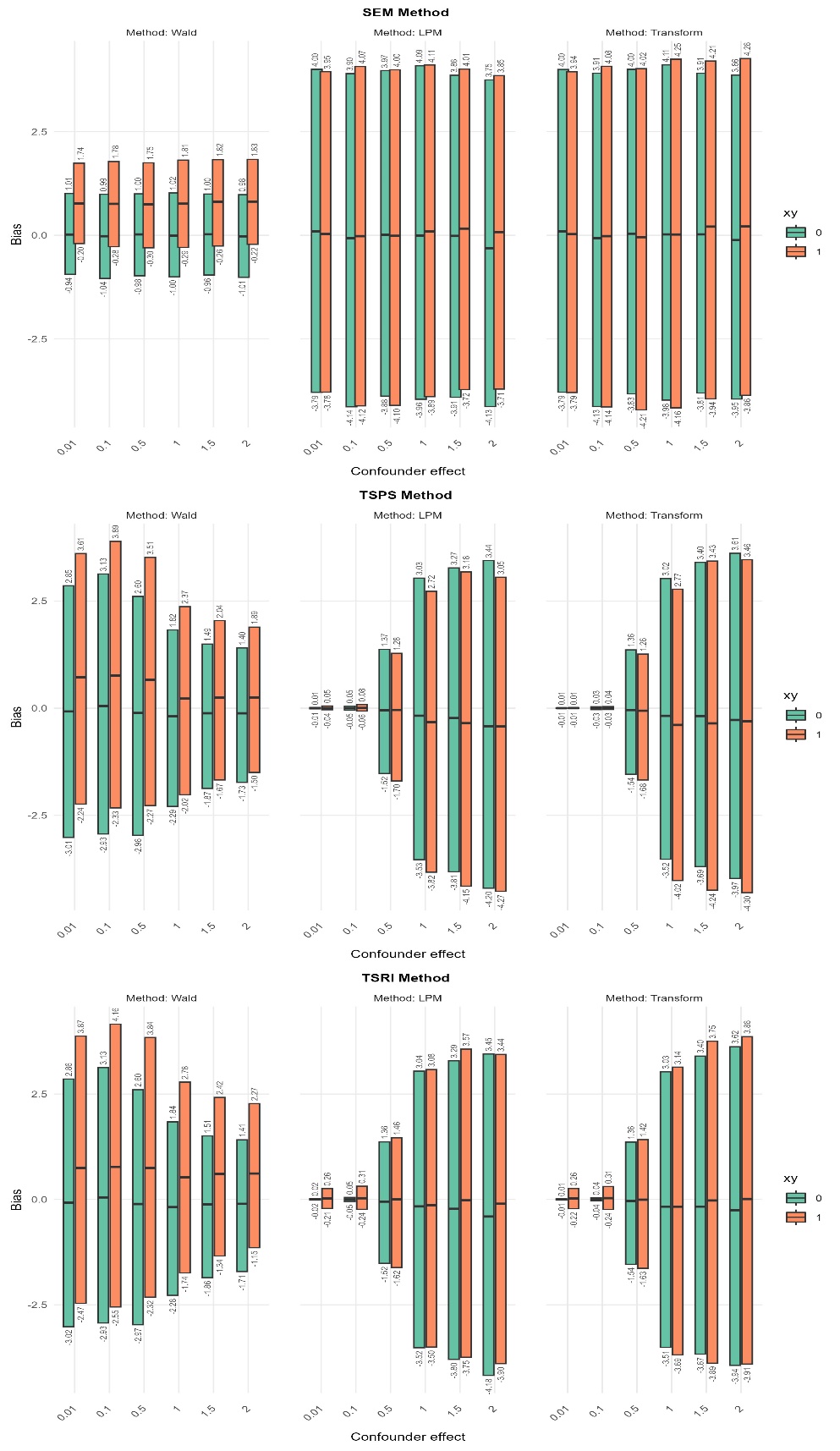


Figure 7: Variation in confounder effects between exposure (cx) and outcome (cy), and the causal effect between exposure and outcome (β₁=xy) set at 0 or 1.

Wald: Wald ratio using logistic regression
LPM: Wald ratio using linear probability model
Transform: Wald ratio using Transformation.

| Scenario | Instrument Strength ($\boldsymbol{\alpha}_{\mathbf{1}}$) | Scenario 1  (Figure 3) | | Scenario 2  (Figure 4) | | Scenario 3  (Figure 5) | | Scenario 4  (Figure 6) | |
| --- | --- | --- | --- | --- | --- | --- | --- | --- | --- |
|  |  | mean X | mean Y | mean X | mean Y | mean X | mean Y | mean X | mean Y |
| β₁(xy)=0 Cx=0.01  Cy=0.01 | 0.01 | 0.50 | 0.50 | 0.73 | 0.73 | 0.95 | 0.95 | 0.12 | 0.12 |
|  | 0.05 | 0.50 | 0.50 | 0.73 | 0.73 | 0.95 | 0.95 | 0.12 | 0.12 |
|  | 0.1 | 0.51 | 0.50 | 0.73 | 0.73 | 0.95 | 0.95 | 0.12 | 0.12 |
|  | 0.5 | 0.52 | 0.50 | 0.75 | 0.73 | 0.96 | 0.95 | 0.13 | 0.12 |
|  | 0.7 | 0.53 | 0.50 | 0.75 | 0.73 | 0.96 | 0.95 | 0.14 | 0.12 |
| β₁(xy)=0 Cx=1.5  Cy=1.5 | 0.01 | 0.50 | 0.50 | 0.67 | 0.67 | 0.90 | 0.90 | 0.19 | 0.19 |
|  | 0.05 | 0.50 | 0.50 | 0.67 | 0.67 | 0.90 | 0.90 | 0.19 | 0.19 |
|  | 0.1 | 0.50 | 0.50 | 0.67 | 0.67 | 0.90 | 0.90 | 0.19 | 0.19 |
|  | 0.5 | 0.52 | 0.50 | 0.69 | 0.67 | 0.91 | 0.90 | 0.20 | 0.19 |
|  | 0.7 | 0.52 | 0.50 | 0.69 | 0.67 | 0.91 | 0.90 | 0.21 | 0.19 |
| β₁(xy)=1 Cx=0.01  Cy=0.01 | 0.01 | 0.50 | 0.62 | 0.73 | 0.84 | 0.95 | 0.98 | 0.12 | 0.14 |
|  | 0.05 | 0.50 | 0.62 | 0.73 | 0.84 | 0.95 | 0.98 | 0.12 | 0.14 |
|  | 0.1 | 0.51 | 0.62 | 0.74 | 0.84 | 0.95 | 0.98 | 0.12 | 0.14 |
|  | 0.5 | 0.52 | 0.62 | 0.75 | 0.84 | 0.96 | 0.98 | 0.13 | 0.14 |
|  | 0.7 | 0.53 | 0.62 | 0.75 | 0.84 | 0.96 | 0.98 | 0.14 | 0.14 |
| β₁(xy)=1  Cx=1.5  Cy=1.5 | 0.01 | 0.50 | 0.58 | 0.67 | 0.75 | 0.90 | 0.94 | 0.19 | 0.23 |
|  | 0.05 | 0.50 | 0.58 | 0.67 | 0.75 | 0.90 | 0.94 | 0.19 | 0.23 |
|  | 0.1 | 0.50 | 0.58 | 0.67 | 0.75 | 0.90 | 0.94 | 0.19 | 0.23 |
|  | 0.5 | 0.52 | 0.58 | 0.69 | 0.75 | 0.91 | 0.94 | 0.20 | 0.23 |
|  | 0.7 | 0.52 | 0.58 | 0.69 | 0.75 | 0.91 | 0.94 | 0.21 | 0.23 |

Table 1: The prevalence of the exposure and outcome for each figure
